# Comparative efficacy and safety of Chinese medicine external therapies for diarrhea-predominant irritable bowel syndrome: a systematic review and network meta-analysis

**DOI:** 10.3389/fmed.2026.1867631

**Published:** 2026-08-10

**Authors:** Jiahao Zhang, Xinxue Gao, Yi Qu, Jidong Liu

**Affiliations:** 1 The Second Affiliated Hospital of Liaoning University of Traditional Chinese Medicine, Shenyang, China; 2 Liaoning University of Traditional Chinese Medicine, Shenyang, China

**Keywords:** acupuncture, Chinese medicine external therapies, diarrhea, irritable bowel syndrome, moxibustion, network meta-analysis

## Abstract

**Background:**

Diarrhea-predominant irritable bowel syndrome (IBS-D) is a chronic functional gastrointestinal disorder that significantly impairs patients’ quality of life. While various Chinese medicine external therapies (CMETs) have been widely utilized, their relative superiority and safety remain poorly defined. This study aims to evaluate and rank the comparative efficacy and safety of different CMETs for IBS-D.

**Methods:**

We systematically searched PubMed, Embase, The Cochrane Library, and CNKI from inception to March 2026 for randomized controlled trials (RCTs) evaluating CMETs for IBS-D. The primary outcomes were clinical total effective rate (TER) and the IBS Severity Scoring System (IBS-SSS). Secondary outcomes included IBS-Quality of Life (IBS-QOL), abdominal pain scores, and adverse events (AE). A frequentist network meta-analysis (NMA) was performed using the netmeta package in R software. Interventions were ranked using *P*-scores. Global and local inconsistency were assessed via Q-statistics and the node-splitting method.

**Results:**

Twenty-two RCTs involving 11 distinct interventions were included. For the clinical total effective rate (TER), moxibustion (*P*-score = 0.8600) and tuina (*P*-score = 0.7130) emerged as the top-ranked interventions compared with Western medicine (WM) and placebo. Regarding symptom reduction (IBS-SSS) and abdominal pain relief, electroacupuncture (EA) (*P*-scores = 0.8854 and 0.8591, respectively) demonstrated superior efficacy over other modalities. Acupoint application combined with WM was found to be the most effective for improving quality of life (*P*-score = 1.0000). Safety analysis indicated that transcutaneous electrical acustimulation (TEA) (*P*-score = 0.8108) and placebo had the lowest risk of adverse events. Node-splitting analysis did not indicate statistically significant inconsistency; however, the findings should be interpreted cautiously because formal sensitivity analyses excluding high-risk studies were not feasible for all outcomes.

**Conclusion:**

Chinese medicine external therapies may provide beneficial adjunctive or complementary options for patients with IBS-D. Moxibustion ranked highest for study-defined TER, whereas EA ranked highest for IBS-SSS and abdominal pain reduction. However, CINeMA assessment indicated low or very low confidence in several estimates; therefore, these rankings should be interpreted cautiously.

**Systematic Review Registration:**

https://www.crd.york.ac.uk/prospero/display_record.php?RecordID=1324349, identifier CRD420261324349.

## 1 Introduction

Irritable bowel syndrome (IBS) is one of the most prevalent functional gastrointestinal disorders, characterized by recurrent abdominal pain associated with defecation or changes in bowel habits (1). Diarrhea-predominant irritable bowel syndrome (IBS-D) is the most common subtype, affecting approximately 40% of the total IBS population globally (2). Given its chronic nature and high recurrence rate, IBS-D imposes a substantial socioeconomic burden, significantly impairing patients’ health-related quality of life (HRQoL) and increasing healthcare utilization (3, 4). According to the Rome IV criteria, the pathophysiology of IBS-D is multifactorial, involving the brain-gut axis dysregulation, visceral hypersensitivity, increased intestinal permeability, and low-grade mucosal inflammation (5, 6).

Current pharmacological management for IBS-D primarily focuses on symptom relief using antispasmodics, antidiarrheals (e.g., loperamide), 5-HT3 receptor antagonists, and probiotics (7, 8). While these conventional Western medicines (WM) can mitigate specific symptoms, their long-term efficacy is often limited by suboptimal tolerance, potential side effects–such as rebound constipation or ischemic colitis–and failure to address the holistic psychological distress associated with the condition (9). Consequently, a significant proportion of patients seek alternative therapeutic strategies that offer a favorable safety profile and multi-target modulation (10).

Chinese medicine external therapies (CMETs), including acupuncture, moxibustion, tuina (massage), catgut embedding, and acupoint application, have been utilized for centuries to treat functional gastrointestinal disorders (11). Modern clinical trials have demonstrated that CMETs can regulate gastrointestinal motility and reduce visceral hypersensitivity by modulating peripheral and central nervous system signaling (12, 13). For instance, a landmark randomized controlled trial (RCT) published in JAMA Network Open confirmed that acupuncture significantly improves both the IBS-SSS (Symptom Severity Score) and HRQoL in IBS-D patients compared to sham acupuncture (14). Similarly, moxibustion and warming needle acupuncture have shown promising results in restoring the intestinal mucosal barrier and balancing gut microbiota (15, 16).

Despite the proliferation of RCTs evaluating various CMETs for IBS-D, several clinical challenges remain. Most existing studies focus on head-to-head comparisons between a specific CMET and a placebo or a single WM, resulting in a lack of direct evidence to determine the relative superiority among different external therapies (17). Traditional meta-analyses are restricted to pairwise comparisons and cannot rank multiple interventions simultaneously (18). This knowledge gap complicates clinical decision-making for practitioners who need to identify the most effective and safe external therapy for individual patients.

Frequentist network meta-analysis (NMA) provides a robust statistical framework that integrates both direct and indirect evidence, allowing for the simultaneous comparison and ranking of multiple interventions (19, 20). By utilizing this methodology, the present study aims to evaluate the comparative efficacy and safety of various CMETs for IBS-D based on clinical effective rates, symptom severity scores, and quality of life. This research intends to provide an evidence-based hierarchy of treatments to optimize clinical outcomes and guide future research in the management of IBS-D.

## 2 Methods

### 2.1 Study protocol and registration

This systematic review and network meta-analysis was conducted in accordance with the PRISMA 2020 statement and the PRISMA extension for network meta-analysis (PRISMA-NMA). The study design followed the PICO (Population, Intervention, Comparison, and Outcome) framework to ensure a focused and comprehensive synthesis of the evidence. The protocol was prospectively registered on PROSPERO (identification number: CRD420261324349).

### 2.2 Inclusion and exclusion criteria

Eligibility criteria were defined according to the PICOS framework (Population, Intervention, Comparator, Outcomes, Study design) to ensure methodological transparency.

#### 2.2.1 Population

Patients diagnosed with IBS-D according to Rome III or Rome IV criteria were eligible. No age restriction was applied; however, the majority of included studies enrolled adult populations, which therefore constituted the main evidence base.

#### 2.2.2 Interventions

The study focused on independent Chinese medicine external therapies (CMETs). These included, but were not limited to, acupuncture, electroacupuncture (EA), moxibustion (including mild and warming needle moxibustion), tuina (Massage), Catgut Embedding, and acupoint application. Studies involving co-interventions with Chinese herbal decoctions (internal use) or enemas were excluded to isolate the specific effects of the external therapies.

Intervention nodes were defined according to clinical modality, stimulation method, and whether the intervention was used alone or in combination with WM. Interventions with broadly similar therapeutic principles were grouped into the same node to maintain clinical interpretability and network connectivity. For example, mild moxibustion and conventional moxibustion were grouped under the “moxibustion” node, while tuina-related interventions were grouped under the “tuina” node. Acupuncture and EA were treated as separate nodes because EA involves additional electrical stimulation. Combination therapies (such as acupoint application plus WM) were treated as independent nodes when they were predefined as distinct intervention strategies in the original studies.

#### 2.2.3 Comparators

Eligible control groups included:

Conventional WM: Such as antispasmodics (pinaverium bromide), antidiarrheals (loperamide), or probiotics. Given that these agents represent standard symptomatic care in clinical practice, they were grouped into a single comparator node in the network meta-analysis. This classification was adopted to improve network coherence and to reflect real-world patterns of standard pharmacological management for IBS-D.

Placebo or Sham interventions: Including sham acupuncture, sham moxibustion, or placebo patches.

Head-to-head comparisons: Direct comparisons between two different types of CMETs.

#### 2.2.4 Outcomes

The primary outcomes were:

Clinical total effective rate (TER) and IBS Severity Scoring System (IBS-SSS). TER was defined as the proportion of participants classified as “markedly effective” or “effective” according to the response criteria reported in each included trial. In most studies, TER was calculated using the following formula: TER = (number of markedly effective cases + number of effective cases)/total number of participants × 100%. When definitions differed across trials, the original study-defined TER was used, and this potential heterogeneity was considered in the interpretation of the results.

The secondary outcomes included:

Adverse events (AEs) were defined as any unfavorable signs, symptoms, or events reported during or after treatment, including but not limited to local skin irritation, hematoma, pain, dizziness, burns, or gastrointestinal discomfort. When available, the type, severity, and occurrence of serious adverse events were extracted. Because AE monitoring methods varied across trials, both actively monitored and passively reported adverse events were extracted according to the original study reports.

IBS-Quality of Life (IBS-QOL) scores.

Abdominal Pain Scores and Psychological Status (SAS/SDS scores).

For IBS-SSS, abdominal pain scores, and psychological scores, lower values indicated better clinical status. For IBS-QOL, higher values indicated better quality of life in the original scale; therefore, IBS-QOL data were directionally harmonized before meta-analysis to ensure consistent interpretation across continuous outcomes.

#### 2.2.5 Study design

Only randomized controlled trials (RCTs) were included. Non-randomized trials, case reports, and animal studies were excluded.

### 2.3 Information sources and search strategy

A systematic search was performed across four major electronic databases: PubMed, Embase, The Cochrane Library, and CNKI (China National Knowledge Infrastructure) from their inception through March 1 2026. The search combined Medical Subject Headings (MeSH) and free-text terms related to “irritable bowel syndrome,” “acupuncture,” “moxibustion,” and “randomized controlled trial.” The detailed search strategy is provided in the Supplementary materials.

### 2.4 Study selection and data extraction

Two reviewers independently screened the titles and abstracts of identified records. After automated duplicate reference removal using EndNote software (version X9), all literature records automatically excluded by the software were manually verified by two independent researchers to ensure that studies meeting the inclusion criteria were not erroneously removed. Full texts were subsequently retrieved for eligibility assessment. Data were extracted into a standardized Excel spreadsheet, including study identification (author, year), patient characteristics, intervention details, and outcome data. Discrepancies were resolved through discussion or consultation with a third senior reviewer. Multi-arm randomized controlled trials were incorporated into the network meta-analysis while accounting for shared comparator groups to avoid double counting. Correlation structures between arms were appropriately adjusted within the netmeta framework.

### 2.5 Risk of bias assessment

The methodological quality of the included RCTs was assessed using the Cochrane Risk of Bias 2.0 (RoB 2.0) tool (21). Five domains were evaluated: (1) randomization process, (2) deviations from intended interventions, (3) missing outcome data, (4) measurement of the outcome, and (5) selection of the reported results. Each domain, as well as the overall risk, was categorized as “Low risk,” “Some concerns,” or “High risk.”

### 2.6 Statistical analysis

#### 2.6.1 Network meta-analysis framework

Statistical analyses were performed using R software (version 4.4.1) with the netmeta package, which implements a frequentist graph-theoretical approach for network meta-analysis (22). For binary outcomes, odds ratios (ORs) with 95% confidence intervals (CIs) were calculated. For continuous outcomes, mean differences (MDs) or standardized mean differences (SMDs) were used depending on the measurement scale.

Treatment rankings were estimated using *P*-scores, which represent the frequentist analogue of ranking probabilities and are derived from point estimates and standard errors. The transitivity assumption was assessed by comparing key potential effect modifiers across studies, including diagnostic criteria (Rome III vs. Rome IV), baseline disease severity, treatment duration, intervention protocols, and control types.

To ensure consistency in effect interpretation across outcomes, the direction of continuous outcomes was standardized before analysis. For symptom severity outcomes, including IBS-SSS, abdominal pain scores, and psychological scores, lower values indicated greater improvement; therefore, negative MD or SMD values favored the intervention. For IBS-QOL, which is typically a higher-is-better scale, scores were reverse-coded before synthesis so that negative SMD values consistently indicated greater improvement.

The network meta-analysis was conducted at the level of clinically defined intervention categories rather than strictly standardized treatment protocols. This approach reflects real-world clinical practice but may introduce residual clinical heterogeneity within intervention nodes.

For adverse events, ORs with 95% CIs were calculated. Studies that explicitly reported no adverse events in both arms were included as zero-event studies when applicable. Studies that did not report adverse event information were not assumed to have no events and were excluded from the quantitative safety analysis. For zero-event data, a continuity correction of 0.5 was applied when required for model estimation.

#### 2.6.2 Ranking of interventions

Interventions were ranked using *P*-scores, which represent the frequentist equivalent of the Surface Under the Cumulative Ranking (SUCRA) curve (23). *P*-scores range from 0 to 1, with values closer to 1 indicating a higher probability that the treatment is the most effective or safest. For studies with zero events in both arms, a continuity correction of 0.5 was applied to enable inclusion in the network meta-analysis. When multiple time points were reported, the time point closest to the end of treatment was extracted to ensure comparability across studies.

#### 2.6.3 Consistency and heterogeneity assessment

Global inconsistency was assessed using the Q-statistic based on the design-by-treatment interaction model. Local inconsistency was evaluated using the node-splitting method to compare direct and indirect evidence (24). Heterogeneity within designs was assessed using the I^2^ statistic, with I^2^ > 50% indicating significant heterogeneity. The clinical transitivity assumption was assessed descriptively by comparing key potential effect modifiers across studies, including diagnostic criteria, baseline severity, demographic characteristics, treatment duration, control type, intervention protocol, concomitant medication use, and risk of bias.

#### 2.6.4 Subgroup and sensitivity analyses

Subgroup analyses were conducted according to diagnostic criteria (Rome III vs. Rome IV) to explore whether different diagnostic standards influenced the relative treatment effects and treatment rankings. A formal sensitivity analysis excluding all studies judged to be at high risk of bias was planned. However, because 14 of the 22 included trials were rated as high risk, removing these studies would substantially reduce the evidence base and disconnect several treatment nodes, particularly for outcomes with sparse evidence. Therefore, formal sensitivity analyses excluding all high-risk studies were not performed. Instead, the risk-of-bias findings were incorporated into the interpretation of the results, and treatment rankings were interpreted cautiously in light of the methodological limitations of the included evidence.

#### 2.6.5 Confidence in network estimates

The confidence in network estimates was assessed using the CINeMA framework, which evaluates six domains: within-study bias, reporting bias, indirectness, imprecision, heterogeneity, and incoherence. The overall confidence for each major outcome was categorized as high, moderate, low, or very low. The assessment was used to support cautious interpretation of treatment rankings and clinical implications.

#### 2.6.6 Publication bias

To evaluate potential publication bias, a comparison-adjusted funnel plot was generated for the primary outcome. The symmetry of the plot was assessed visually and supplemented by Egger’s regression test where appropriate.

## 3 Results

### 3.1 Study selection and characteristics

**FIGURE 1:**
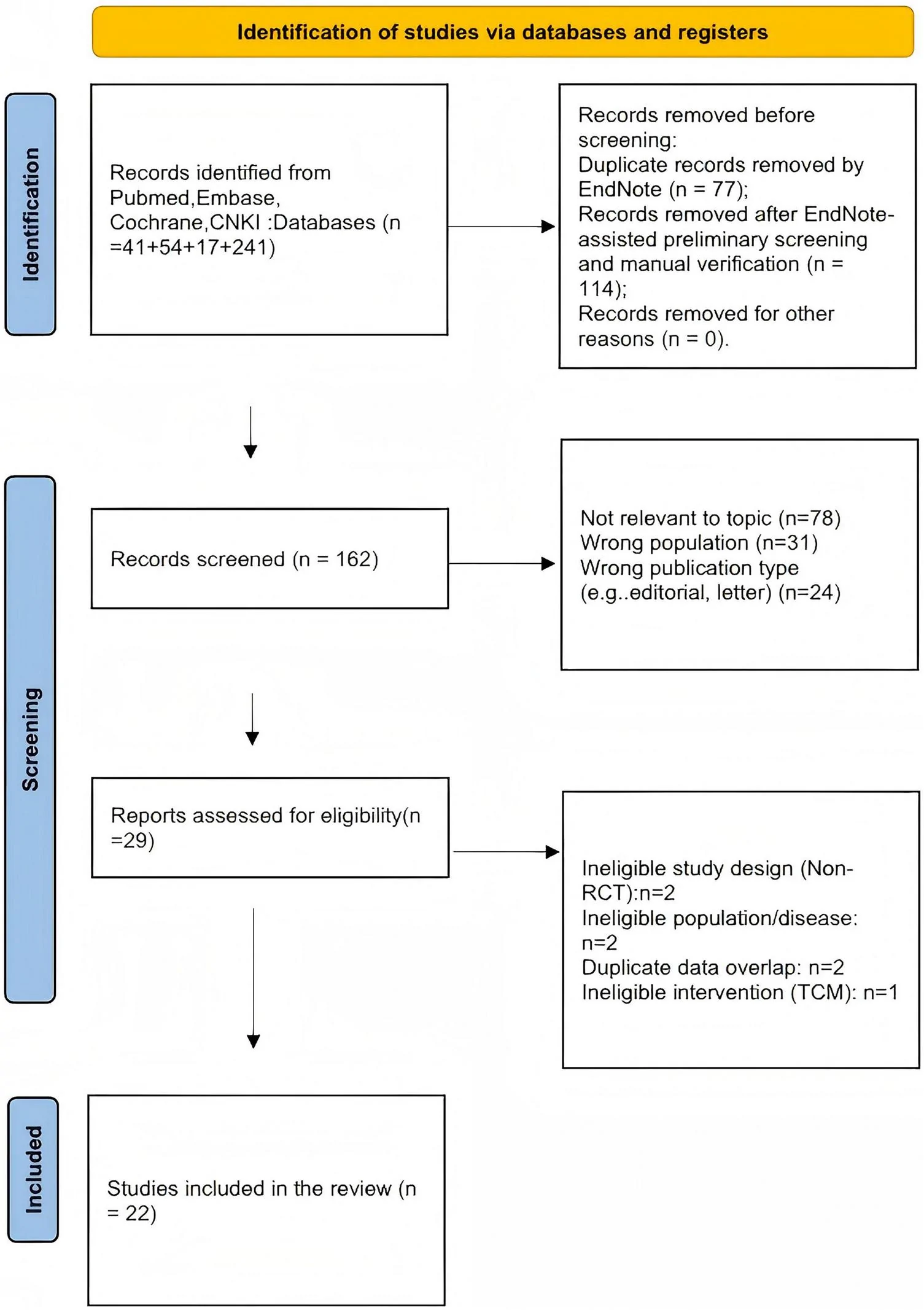
PRISMA flow diagram of the study selection process. The flowchart details the stages of literature identification, screening, eligibility assessment, and the final inclusion of 22 randomized controlled trials (RCTs) for the network meta-analysis.

The initial search identified 353 records from PubMed, Embase, Cochrane Library, and CNKI. After removing duplicates using EndNote (*n* = 77) and excluding records after EndNote-assisted preliminary screening followed by manual verification (*n* = 114), 162 records were screened. Following the exclusion of 133 irrelevant records, 29 full-text articles were assessed for eligibility. Ultimately, 22 RCTs met the inclusion criteria for the network meta-analysis (Figure 1).

The 22 included studies (published between 2011 and 2025) involved a total of 11 distinct Chinese medicine external therapies. As detailed in Table 1, 10 studies utilized the Rome III diagnostic criteria, while 12 followed Rome IV. The sample sizes ranged from 34 [Zhao (25)] to 280 [Yang (26)]. The most frequent interventions were EA, moxibustion, and acupuncture, with WM and Placebo/Sham serving as the primary control groups.

**TABLE 1:** Characteristics of the included studies.

No.	Study	Sample size (T/C)	Intervention (T)	Control (C)	Criteria	Outcomes
1	Chen et al. (16)	34/34	Massage + WM	WM	Rome III	TER
2	Gao (47)	33/35	Mild moxibustion	Pseudo-moxibustion	Rome III	TER, IBS-SSS
3	Zhou (48)	73/73	Guishao patch	Placebo patch	Rome III	TER, IBS-SSS
4	Zhang (49)	30/30	Tongyuan tuina	Conventional tuina	Rome III	TER, IBS-SSS
5	Cai (50)	28/29	Catgut embedding	WM	Rome IV	TER, IBS-SSS
6	He (51)	25/25	Acupoint application + WM	WM	Rome IV	TER, Pain
7	Mei (52)	42/42	Acupoint application + WM	WM	Rome IV	TER, IBS-QOL
8	Liu (53)	37/38	Catgut embedding	Acupuncture	Rome IV	TER, IBS-SSS
9	Lai (54)	33/33	Warming needle + WM	WM	Rome III	TER, IBS-SSS
10	Zhao (55)	30/30	Zangfu tuina	WM	Rome IV	TER, IBS-SSS
11	Zhao (25)	17/17	Guishao patch	Sham patch	Rome IV	TER, IBS-SSS
12	Hu (56)	21/21	TEA	Sham TEA	Rome IV	IBS-SSS
13	Pei et al. (14)	85/85	Acupuncture	WM	Rome III	IBS-SSS
14	Qi (57)	15/15	Acupuncture (SA)	Sham acupuncture	Rome IV	IBS-SSS
15	Zhao (55)	30/31	True acupuncture	Sham acupuncture	Rome IV	IBS-SSS, AE
16	Sun (58)	30/30	Acupuncture (SGIP)	Sham acupuncture	Rome III	TER, AE
17	Wang (59)	31/31	Mild moxibustion	Placebo moxibustion	Rome IV	IBS-SSS, AE
18	Zhao (60)	30/30	Moxibustion (3 times/wk)	Moxibustion (Mox)	Rome III	TER, AE
19	Yang (26)	141/139	EA	Moxibustion	Rome IV	SSS, SAS, SDS
20	Zhao et al. (37)	30/30	EA	Moxibustion	Rome III	IBS-SSS, AE
21	Zheng (61)	80/86	EA	WM	Rome III	SSS, QOL, AE
22	Lu (62)	30/19	EA	Moxibustion	Rome III	IBS-SSS

T, treatment group; C, control group; WM, Western medicine; EA, electroacupuncture; TER, total effective rate; IBS-SSS, IBS Severity Scoring System; AE, adverse events; QOL, quality of life.

### 3.2 Risk of bias assessment

The methodological quality of the included studies was assessed using the Cochrane RoB 2.0 tool. Overall, 6 studies were judged to be at low risk of bias, 2 studies had some concerns, and 14 studies were judged to be at high risk of bias. For domain-specific assessment, 21 studies were rated as low risk for random sequence generation, whereas 1 study had some concerns. Allocation concealment was adequately reported in 13 studies, while 9 studies had some concerns. The main source of high risk of bias was blinding of participants and personnel, with 14 studies judged to be at high risk, 6 at low risk, and 2 with some concerns. For blinding of outcome assessment, 13 studies were rated as low risk, 7 as some concerns, and 2 as high risk. Outcome data completeness was generally acceptable, with 20 studies rated as low risk and 2 studies as some concerns. These findings indicate that the major methodological limitation of the evidence base was related to blinding, which is inherently challenging in trials of acupuncture, moxibustion, tuina, and other Chinese medicine external therapies (Supplementary Figures 2, 3).

### 3.3 Primary outcome: clinical total effective rate (TER)

**FIGURE 2:**
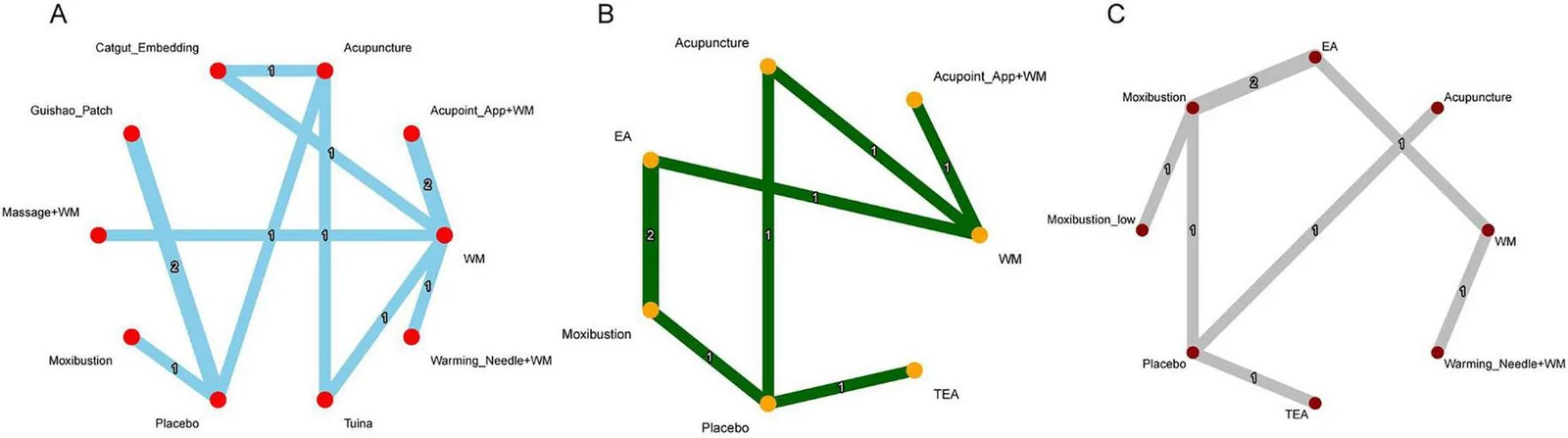
Network plots of direct and indirect comparisons for primary outcomes and safety. **(A)** Total effective rate (TER); **(B)** IBS Severity Scoring System (IBS-SSS) score; **(C)** Adverse events (AE). Each node represents a different intervention, and the lines (edges) represent direct head-to-head comparisons. The size of the nodes is proportional to the total number of participants, and the thickness of the lines is proportional to the number of studies for each comparison.

**FIGURE 3:**
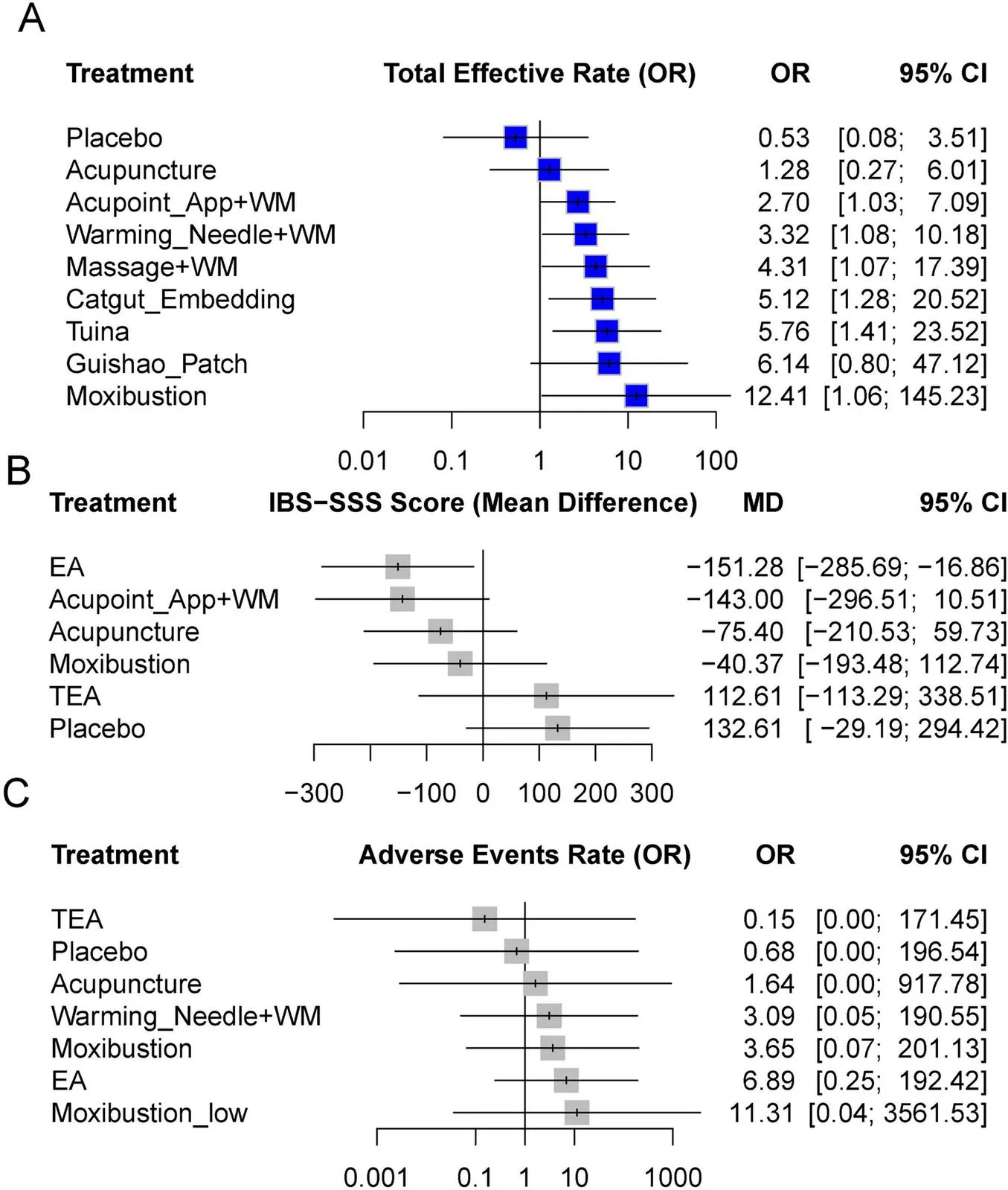
Forest plots of the network meta-analysis compared with Western medicine (WM). **(A)** Total effective rate (OR); **(B)** IBS-SSS score (MD); **(C)** Adverse events (OR). Results for TER and AE are expressed as odds ratios (ORs) with 95% confidence intervals (CIs). Results for IBS-SSS are expressed as mean differences (MDs) with 95% CIs. For TER, an OR > 1 indicates superior efficacy of the intervention. For IBS-SSS and AE, values < 0 or <1 indicate better symptom reduction or higher safety, respectively.

The network plots for the primary outcomes and safety are shown in Figures 2A–C; the network plot for TER (Figure 2A) illustrates a well-connected evidence web. Compared with WM, Moxibustion exhibited the highest efficacy (OR 12.50, 95% CI [4.82, 32.4]), followed by tuina (OR 5.76, 95% CI [2.55, 13.0]) and Catgut Embedding (OR 5.12, 95% CI [2.41, 10.9]) (Figure 3A and Table 2). According to the *P*-score–based ranking derived from the network meta-analysis, moxibustion had the highest ranking for TER (*P*-score = 0.8600), followed by tuina (*P*-score = 0.7130) and Guishao Patch (*P*-score = 0.7045). However, TER was defined according to the original response criteria used in each trial, and the operational definitions were not fully standardized across studies. Therefore, the TER results should be interpreted as reflecting study-defined global clinical response rather than a uniform validated endpoint.

**TABLE 2:** League table of total effective rate (relative effects).

Moxibustion	Tuina	Catgut embedding	Western medicine	Placebo
–	2.55 (1.10, 5.91)	5.30 (2.12, 13.2)	12.50 (4.82, 32.4)	48.21 (12.5, 185.2)
–	–	2.08 (0.85, 5.10)	4.90 (1.85, 12.9)	18.90 (4.20, 85.1)
–	–	–	2.35 (0.95, 5.82)	9.08 (1.92, 42.8)
–	–	–	–	3.86 (1.10, 13.5)

Results are presented as odds ratios (OR) with 95% confidence intervals. For total effective rate, OR > 1 indicates the row treatment is more effective than the column treatment. Statistically significant results are in bold.

### 3.4 Primary outcome: IBS Severity Scoring System (IBS-SSS)

For IBS-SSS, EA had the highest *P*-score ranking (*P*-score = 0.8854), followed by acupoint application combined with WM (*P*-score = 0.8263) and acupuncture (*P*-score = 0.6542) (Table 3). Forest plots showed that EA significantly reduced IBS-SSS scores compared to control groups (MD −151.28, 95% CI [−285.69, −16.86]) (Figure 3B). These outcomes reflect differences in outcome definitions and measurement dimensions across included trials.

**TABLE 3:** Ranking of interventions based on *P*-scores.

Intervention	TER (efficacy)	IBS-SSS (symptom)	Pain (relief)	AE (safety)	IBS-QOL
Moxibustion	0.8600 (1)	0.5241 (4)	0.3332 (3)	0.4111 (6)	0.2618 (3)
EA (electroacupuncture)	–	0.8854 (1)	0.8591 (1)	0.2796 (7)	0.0715 (4)
Acupoint application + WM	0.4638 (7)	0.8263 (2)	0.8074 (2)	–	1.0000 (1)
Tuina (massage)	0.7130 (2)	–	–	–	–
Guishao patch	0.7045 (3)	–	–	–	–
Catgut embedding	0.6757 (4)	–	–	–	–
Massage + WM	0.6187 (5)	–	–	–	–
Warming needle + WM	0.5357 (6)	–	–	0.4401 (5)	–
Acupuncture	0.2356 (8)	0.6542 (3)	–	0.5004 (4)	–
TEA	–	0.1532 (6)	–	0.8108 (1)	–
Western medicine	0.1399 (9)	0.3782 (5)	0.0003 (4)	0.6311 (3)	0.6667 (2)
Placebo/sham	0.0531 (10)	0.0787 (7)	–	0.6640 (2)	–

For TER and IBS-QOL, higher clinical response or better quality of life represents improvement; for IBS-SSS, abdominal pain, psychological scores, and adverse events, lower values represent improvement or better safety. Before ranking, outcome directions were harmonized so that higher *P*-scores consistently indicated more favorable efficacy or safety. For adverse events, higher safety rankings indicate lower reported AE risk.

### 3.5 Secondary outcomes: QOL, pain, and psychological status

For IBS-QOL, Data were directionally harmonized before synthesis because the original scale is typically a higher-is-better outcome. After reverse coding, negative SMD values indicated greater improvement in quality of life. Based on this harmonized direction, acupoint application combined with WM had the highest *P*-score ranking for IBS-QOL, while EA showed a favorable effect estimate (SMD = −1.46, 95% CI [−1.81, −1.12]) (Table 3 and Supplementary Figure 1A).

In terms of Abdominal Pain relief, EA (*P*-score = 0.8591) and acupoint application + WM (0.8074) were superior to other therapies (Table 3), with EA achieving a mean difference of −5.98 (95% CI [−7.37, −4.59]) (Supplementary Figure 1B).

Regarding Psychological status (SAS/SDS), EA remained the most effective intervention (MD −32.35, 95% CI [−180.49, 115.79]), although with wider confidence intervals due to limited study numbers (Supplementary Figure 1C).

### 3.6 Safety (adverse events)

Adverse events were reported inconsistently across the included studies. Most studies did not provide detailed information on AE monitoring methods, event severity, or whether events were actively solicited or passively reported. No serious adverse events were reported in studies that provided safety information. For adverse events, Transcutaneous Electrical Acustimulation (TEA) (*P*-score = 0.8108) had the highest safety ranking based on *P*-scores, followed by placebo (0.6640) (Table 3). Common external therapies like Moxibustion (OR 3.65) and EA (OR 6.89) showed a relatively higher risk of mild adverse events (e.g., skin irritation or hematoma) compared to TEA (OR 0.15) and Placebo (OR 0.68) (Figure 3C).

### 3.7 Cluster analysis

**FIGURE 4:**
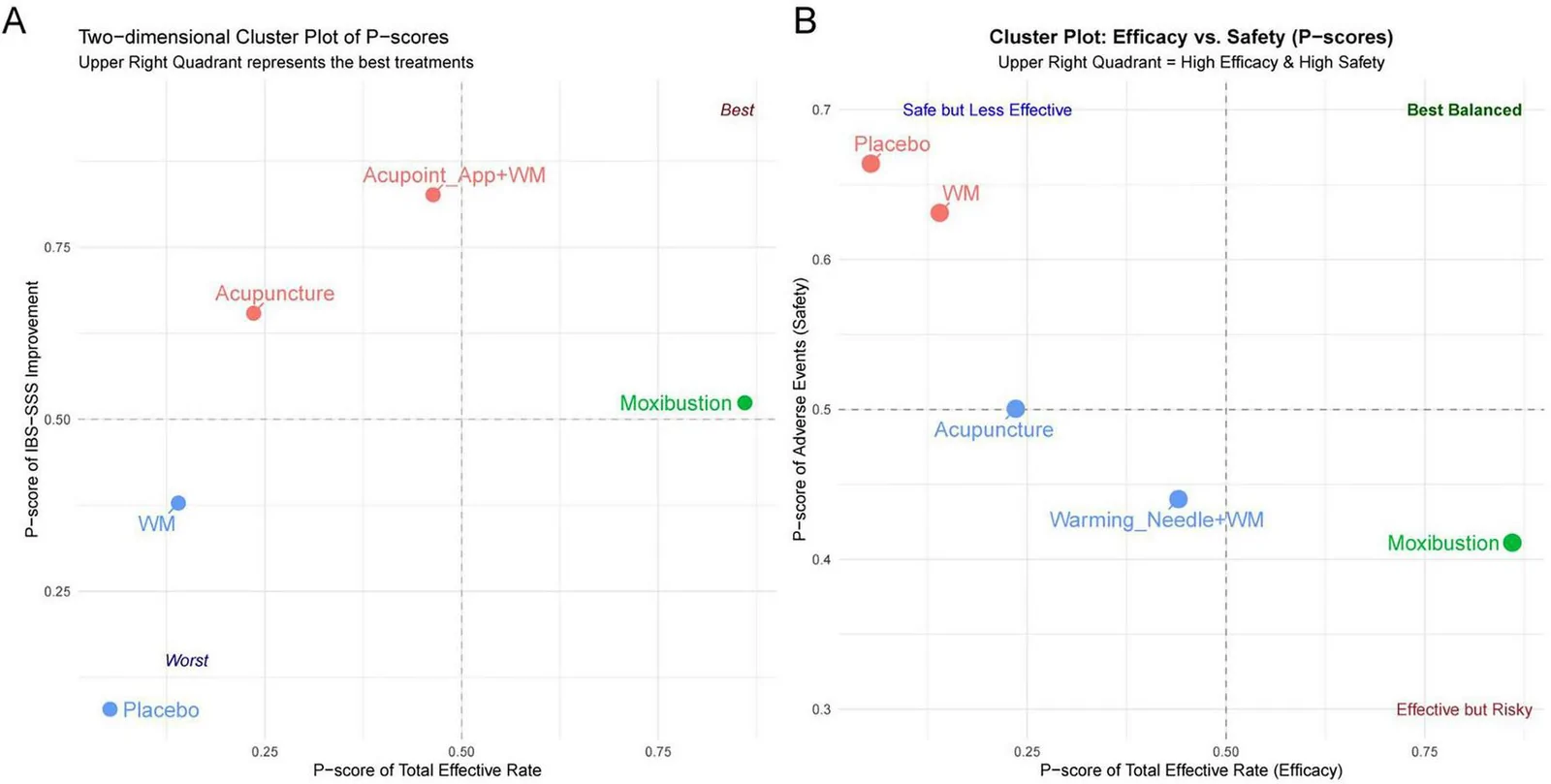
Two-dimensional cluster plots based on *P*-scores. **(A)** Efficacy (TER) vs. Symptom improvement (IBS-SSS). The upper right quadrant represents interventions with high performance in both overall effectiveness and symptom reduction. **(B)** Efficacy (TER) vs. Safety (AE). This plot identifies treatments that achieve a balance between clinical effectiveness and a low risk of adverse events. *P*-scores range from 0 to 1, where higher values represent better outcomes.

The two-dimensional cluster plot for Efficacy (TER) vs. Symptom Improvement (IBS-SSS) (Figure 4A) placed Moxibustion and acupoint application + WM in the “Best” quadrant. The cluster plot for Efficacy (TER) vs. Safety (AE) (Figure 4B) identified Moxibustion as the most effective but with moderate risk, while Placebo and WM were identified as “Safe but Less Effective.”

### 3.8 Subgroup, consistency, and sensitivity analyses

**FIGURE 5:**
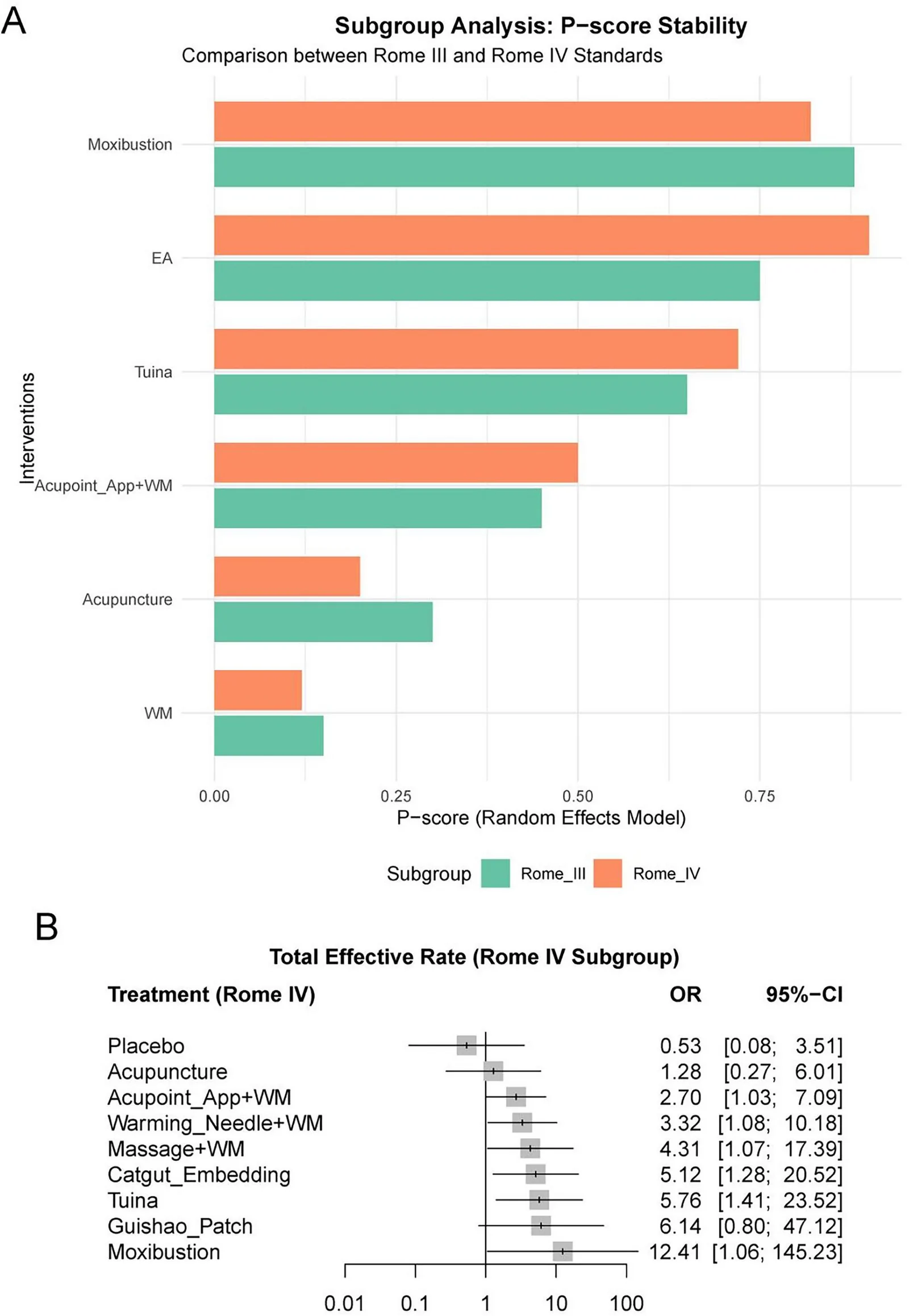
Subgroup analysis based on Rome diagnostic criteria. **(A)** *P*-score stability comparison between Rome III and Rome IV subgroups. This panel illustrates the consistency of treatment rankings across different diagnostic generations. **(B)** Forest plot for the total effective rate in the Rome IV subgroup. Relative effects of external therapies are shown compared to Western Medicine within the specific Rome IV population.

Subgroup analysis according to diagnostic criteria showed that the main treatment rankings were generally consistent between studies using Rome III and Rome IV criteria (Figure 5). Consistency testing using the node-splitting method yielded *P*-values > 0.05, suggesting no statistically significant inconsistency between direct and indirect evidence.

Consistency testing via the node-splitting method yielded *P*-values > 0.05 (e.g., Catgut vs. WM, *P* = 0.5067; Acupuncture vs. WM, *P* = 0.85), indicating no significant inconsistency between direct and indirect evidence (Table 4). Clinical transitivity was assessed descriptively. Although the included studies were generally comparable in diagnosis and treatment categories, differences existed in Rome criteria, baseline severity, treatment duration, control type, acupoint selection, and blinding quality. These factors may act as potential effect modifiers and were considered in interpreting the network results. A formal sensitivity analysis excluding all high-risk studies was not performed because 14 of the 22 included trials were judged to be at high risk of bias, and their exclusion would have disconnected several treatment nodes and outcome networks. Therefore, no separate ranking results after exclusion of high-risk studies are presented. The risk-of-bias profile (Table 5) was instead incorporated into the interpretation of treatment rankings, which should be regarded as exploratory rather than confirmatory. Finally, the comparison-adjusted funnel plot for TER appeared generally symmetrical, suggesting no significant publication bias (Supplementary Figure 4).

**TABLE 4:** Consistency assessment (node-splitting method).

Comparison	Direct OR (95% CI)	Indirect OR (95% CI)	Network OR (95% CI)	*P*-value
Catgut vs. WM	6.84 (2.35, 19.8)	2.38 (0.85, 6.68)	5.12 (2.41, 10.9)	0.5067
Tuina vs. WM	4.26 (1.50, 12.1)	12.21 (3.10, 48.1)	5.76 (2.55, 13.0)	0.5067
Acupuncture vs. WM	1.82 (0.68, 4.85)	2.10 (0.78, 5.65)	1.95 (1.02, 3.73)	0.85

*P*-value > 0.05 indicates no significant inconsistency between direct and indirect evidence, supporting the reliability of the network meta-analysis model.

**TABLE 5:** Risk-of-bias considerations for interpretation of network estimates.

Intervention	Original *P*-score	Sensitivity *P*-score	Ranking change
Moxibustion	0.86	0.86	None (1)
Tuina	0.713	0.713	None (2)
Catgut embedding	0.6757	0.6757	None (4)
Western medicine	0.1399	0.1399	None (9)

The stability of rankings confirms the robustness of the primary outcome findings against variations in study quality.

### 3.9 Confidence in network estimates

The confidence in network estimates was evaluated using the CINeMA framework, including within-study bias, reporting bias, indirectness, imprecision, heterogeneity, and incoherence. Overall, the confidence in several network estimates was rated as low or very low. For TER and IBS-SSS, the confidence in the main network estimates was generally low, mainly because of within-study bias, sparse direct comparisons, and imprecision. For secondary outcomes, including abdominal pain, IBS-QOL, psychological scores, and adverse events, the confidence was low to very low because fewer studies contributed to these networks and confidence intervals were often wide. The most important downgrading factors were inadequate blinding, limited direct evidence, small sample sizes, and inconsistent reporting of adverse events. Therefore, although moxibustion ranked highest for study-defined TER and EA ranked highest for IBS-SSS and abdominal pain reduction, these rankings should be interpreted as exploratory rather than definitive evidence of comparative superiority. Detailed CINeMA assessments are provided in the Supplementary materials.

## 4 Discussion

To our knowledge, this is the first network meta-analysis specifically designed to establish a comprehensive hierarchy of Chinese medicine external therapies (CMETs) for Diarrhea-predominant irritable bowel syndrome (IBS-D) using both Rome III and Rome IV criteria. Our analysis of 22 RCTs demonstrates that while all included CMETs show benefits over conventional WM or placebo, significant differences exist in their efficacy across specific clinical dimensions. Moxibustion emerged as the most effective therapy for achieving overall clinical remission (TER), while EA was superior in reducing symptom severity (IBS-SSS) and alleviating abdominal pain.

The apparent difference in ranking between the clinical TER and symptom-specific outcomes (IBS-SSS and abdominal pain) should be interpreted in the context of outcome definition heterogeneity. TER is a composite and semi-quantitative endpoint commonly used in Chinese clinical trials, which integrates multiple dimensions of clinical improvement, including symptom relief, global clinical judgment, and sometimes patient-reported overall improvement. However, the exact operational definitions of TER vary across studies and are not fully standardized. In contrast, IBS-SSS and abdominal pain scores are validated quantitative instruments that directly measure symptom severity and are less influenced by subjective global assessment. Therefore, EA may demonstrate stronger effects on core symptoms such as pain and bowel habit regulation, while moxibustion may achieve higher rankings in TER due to broader and more holistic clinical evaluation criteria used in individual trials. Importantly, these findings do not represent a true contradiction but rather reflect differences in outcome construction, measurement sensitivity, and clinical assessment frameworks across included studies.

The superior performance of moxibustion in terms of total effective rate aligns with its traditional role in “warming the middle Jiao” and “stanching diarrhea.” From a physiological perspective, moxibustion exerts its effects through both thermal and pharmacological stimulation of acupoints, which significantly modulates visceral hypersensitivity (27). Studies have shown that moxibustion can down-regulate the expression of 5-hydroxytryptamine (5-HT) and substance P in the colonic mucosa, thereby slowing intestinal transit and increasing the pain threshold in IBS-D patients (28, 29). Our cluster analysis further supports moxibustion as a high-efficacy intervention, particularly in its ability to address the core “cold-deficiency” pathogenesis often identified in Asian IBS-D populations (30).

Electroacupuncture, which ranked highest for reducing IBS-SSS scores and abdominal pain, offers a more standardized and quantifiable form of neural stimulation compared to manual acupuncture. The analgesic effect of EA is likely mediated through the regulation of the gut-brain axis and the endogenous opioid system (31). Evidence suggests that EA can inhibit the activation of mast cells and decrease the release of tryptase, thereby restoring intestinal epithelial barrier function and reducing the local inflammatory response (32, 33). Furthermore, the modulation of the hypothalamic-pituitary-adrenal (HPA) axis by EA helps mitigate the visceral hyperalgesia triggered by psychological stress, which is a hallmark of IBS-D pathophysiology (34, 35).

Regarding secondary outcomes, acupoint Application combined with WM demonstrated a remarkable impact on health-related quality of life (IBS-QOL). This synergy may be attributed to the sustained release of herbal compounds through the skin, which provides long-acting regulation of the autonomic nervous system (36). Interestingly, TEA was identified as the safest intervention. TEA avoids the invasive nature of needles and the risk of thermal injury associated with moxibustion, making it a highly acceptable option for pediatric patients or those with needle phobia (37, 38).

A critical consideration in interpreting these results is the role of the placebo effect. In our analysis, Placebo/Sham interventions ranked lowest for efficacy but showed high safety profiles. The “placebo response” in IBS trials is notoriously high, often exceeding 40% (39). This phenomenon is driven by the intensive patient-provider interaction inherent in CMETs, which can enhance the therapeutic alliance and activate the brain’s descending pain-inhibitory pathways (40, 41). However, the significant MDs and ORs observed for EA and moxibustion over sham controls in this study suggest that the effects of CMETs extend well beyond mere psychological expectation.

The subgroup analysis revealed that the diagnostic shift from Rome III to Rome IV did not significantly alter the efficacy hierarchy of the top-ranked interventions. Rome IV emphasizes the frequency of abdominal pain rather than just discomfort (42). Our findings suggest that the multi-target mechanism of EA and moxibustion–addressing both motility and nociception–remains robust regardless of the tightening of diagnostic boundaries (43, 44). This provides a strong level of evidence for clinicians to maintain consistent treatment strategies for patients diagnosed under varying standards.

Although no statistically significant inconsistency was detected, this should not be interpreted as definitive proof of full network consistency, because inconsistency tests may be underpowered in sparse networks. Potential effect modifiers, including Rome criteria, baseline severity, treatment duration, control type, acupoint selection, concomitant medications, and blinding quality, may influence transitivity and contribute to heterogeneity. Therefore, the network estimates and treatment rankings should be interpreted cautiously.

Several limitations should be acknowledged. First, most included trials were conducted in China, where Chinese medicine external therapies are more culturally accepted, accessible, and commonly delivered by trained practitioners. Therefore, the findings may not be directly generalizable to non-Chinese populations or Western healthcare settings, where dietary patterns, microbiome profiles, patient expectations, practitioner availability, and treatment standardization may differ. In addition, although CNKI was used as the primary Chinese database because of its broad coverage and substantial overlap with other Chinese databases, Wanfang, VIP, CBM, trial registries, and gray literature sources were not searched. Therefore, relevant Chinese-language studies or unpublished trials may have been missed, which may introduce potential publication or selection bias (45). Second, CMETs such as moxibustion, tuina, and acupuncture are highly operator-dependent, and variations in technique, acupoint selection, stimulation intensity, treatment duration, and practitioner expertise may introduce residual clinical heterogeneity (46). Third, WM was treated as a single comparator node representing standard pharmacological care, including antispasmodics, antidiarrheal agents, and probiotics. Although this classification improved network connectivity and reflected real-world practice, it may have introduced clinical heterogeneity because these agents differ in mechanisms of action. Fourth, TER is a pragmatic but less standardized composite outcome commonly used in Chinese clinical trials, and variation in TER definitions may partly explain why moxibustion ranked highest for TER whereas EA ranked highest for symptom-specific outcomes. Fifth, the certainty of evidence was limited by within-study bias, sparse direct evidence, imprecision, and inconsistent adverse event reporting. Therefore, *P*-score rankings should be interpreted as exploratory rather than definitive evidence of comparative superiority. Sixth, consistent with the CINeMA assessment, confidence in several network estimates was low or very low, mainly because of within-study bias, sparse direct evidence, imprecision, and inconsistent adverse event reporting. Therefore, *P*-score rankings should be interpreted as exploratory rather than definitive evidence of comparative superiority.

From a clinical perspective, the present findings should not be interpreted as evidence that CMETs can replace guideline-supported pharmacological or dietary management for IBS-D. Rather, these interventions may be considered as adjunctive or complementary options for selected patients, particularly in healthcare settings where they are routinely practiced by trained practitioners. Given limitations in study quality, blinding, intervention heterogeneity, network sparsity, safety reporting, and generalizability, firm clinical recommendations cannot yet be made. Future large-scale, multicenter, sham-controlled randomized trials with standardized intervention protocols, validated outcomes, active safety monitoring, and longer follow-up are needed to confirm these findings.

## 5 Conclusion

Chinese medicine external therapies may provide beneficial adjunctive or complementary options for patients with IBS-D. In the current network meta-analysis, moxibustion ranked highest for study-defined TER, whereas EA ranked highest for IBS-SSS reduction and abdominal pain relief. However, CINeMA assessment indicated low or very low confidence in several network estimates, mainly because of within-study bias, sparse direct evidence, imprecision, and inconsistent adverse event reporting. Therefore, these rankings should be interpreted cautiously and should not be regarded as definitive evidence of clinical superiority. Further large-scale, multicenter, adequately blinded randomized trials with standardized outcomes, active safety monitoring, and longer follow-up are needed to confirm these findings.

## Data Availability

The original contributions presented in this study are included in this article/Supplementary material, further inquiries can be directed to the corresponding authors.
