# Supplementary material for: Comparative efficacy and safety of Chinese medicine external therapies for diarrhea-predominant irritable bowel syndrome: a systematic review and network meta-analysis
Source: Front Med (Lausanne). 2026 Aug 10;13:1867631. doi: 10.3389/fmed.2026.1867631 (PMC13500253; doi:10.3389/fmed.2026.1867631)
Supplement: Supplementary file 1 [file Data_Sheet_1.pdf]

Supplementary Materials

**Pubmed**	
Step	Search Terms
#1	("Irritable Bowel Syndrome"[Mesh] OR "Irritable Bowel Syndrome"[Title/Abstract] OR "IBS"[Title/Abstract] OR "IBS-D"[Title/Abstract] OR "diarrhea-predominant irritable bowel syndrome"[Title/Abstract])
#2	("Medicine, Chinese Traditional"[Mesh] OR "Acupuncture Therapy"[Mesh] OR "Moxibustion"[Mesh] OR "Massage"[Mesh] OR "acupuncture"[Title/Abstract] OR "electroacupuncture"[Title/Abstract] OR "moxibustion"[Title/Abstract] OR "warming needle"[Title/Abstract] OR "catgut embedding"[Title/Abstract] OR "acupoint application"[Title/Abstract] OR "tuina"[Title/Abstract] OR "acupressure"[Title/Abstract])
#3	("randomized controlled trial"[Publication Type] OR "randomized"[Title/Abstract] OR "placebo"[Title/Abstract])
Final	#1 AND #2 AND #3
**Embase**	
('irritable bowel syndrome'/exp OR 'irritable bowel syndrome':ti,ab OR IBS:ti,ab OR IBS-D:ti,ab) AND ('acupuncture'/exp OR 'moxibustion'/exp OR 'traditional chinese medicine'/exp OR acupuncture:ti,ab OR electroacupuncture:ti,ab OR moxibustion:ti,ab OR tuina:ti,ab OR 'catgut embedding':ti,ab OR 'acupoint application':ti,ab) AND ('randomized controlled trial'/exp OR randomized:ti,ab OR placebo:ti,ab)	
**Cochrane Library**	
(irritable bowel syndrome OR IBS OR IBS-D):ti,ab,kw AND (acupuncture OR moxibustion OR electroacupuncture OR tuina OR "catgut embedding" OR "acupoint application"):ti,ab,kw AND (randomized OR placebo):ti,ab,kw	
**CNKI**	
主题 =("肠易激综合征" OR "腹泻型肠易激综合征" OR IBS) AND 主题 =("针灸" OR "电针" OR "艾灸" OR "推拿" OR "穴位贴敷" OR "埋线") AND 主题 =("随机" OR "随机对照" OR "临床试验")	

Appendix A. Detailed Search Strategy (PubMed)

EA	--	--	--	--
-38.45 (-75.1, -1.8)	Acupuncture	--	--	--
-52.10 (-98.2, -6.0)	-13.65 (-55.3, 28.0)	Moxibustion	--	--
-108.4 (-145.2, -71.6)	-69.95 (-95.8, -44.1)	-56.30 (-95.5, -17.1)	Western Med.	--
-156.2 (-198.5, -113.9)	-117.7 (-158.3, -77.1)	-104.1 (-148.9, -59.3)	-47.80 (-85.2, -10.4)	Placebo

Appendix B. League Table for IBS-SSS (Secondary Outcome)

TEA	0.82 (0.15, 4.45)	0.65 (0.10, 4.21)	0.52 (0.08, 3.32)	0.22 (0.04, 1.25)
--	Placebo	0.79 (0.28, 2.21)	0.63 (0.21, 1.91)	0.27 (0.09, 0.82)
--	--	Western Med.	0.80 (0.22, 2.95)	0.34 (0.10, 1.15)
--	--	--	Acupuncture	0.42 (0.11, 1.62)
--	--	--	--	EA

Appendix C. League Table for Adverse Events (Safety Outcome)

Total Heterogeneity/Inconsistency (Q): 0.50 (df = 3), p-value = 0.9197
Within-design Heterogeneity: 0.06 (df = 2), p-value = 0.9726
Between-design Inconsistency: 0.44 (df = 1), p-value = 0.5067

Net Split Result: The p-values for all node-splitting comparisons were > 0.05, suggesting that the "Transitivity" assumption of the network was satisfied.

Appendix D. Heterogeneity and Global Inconsistency Test

Outcome	Main finding	RoB concern	Imprecision	Incoherence	Overall confidence
TER	Moxibustion ranked highest	Serious	Moderate	Not serious	Low
IBS-SSS	EA ranked highest	Serious	Serious	Not serious	Low
Pain	EA ranked highest	Serious	Serious	Not serious	Low/Very low
IBS-QOL	Acupoint application + WM ranked highest	Serious	Serious	Not serious	Very low
AE	TEA ranked highest for safety	Serious	Serious	Not serious	Very low

Appendix E. CINeMA assessment of confidence in network estimates.

Study	Diagnostic criteria	TER definition	Response categories	Time point	Consistency
Chen 2018	Rome III	Study-defined clinical response	Markedly effective/effective/ineffective	End of treatment	Partially consistent
Gao 2020	Rome III	Study-defined symptom improvement	Markedly effective/effective/ineffective	End of treatment	Partially consistent

Appendix F. study-defined response criteria

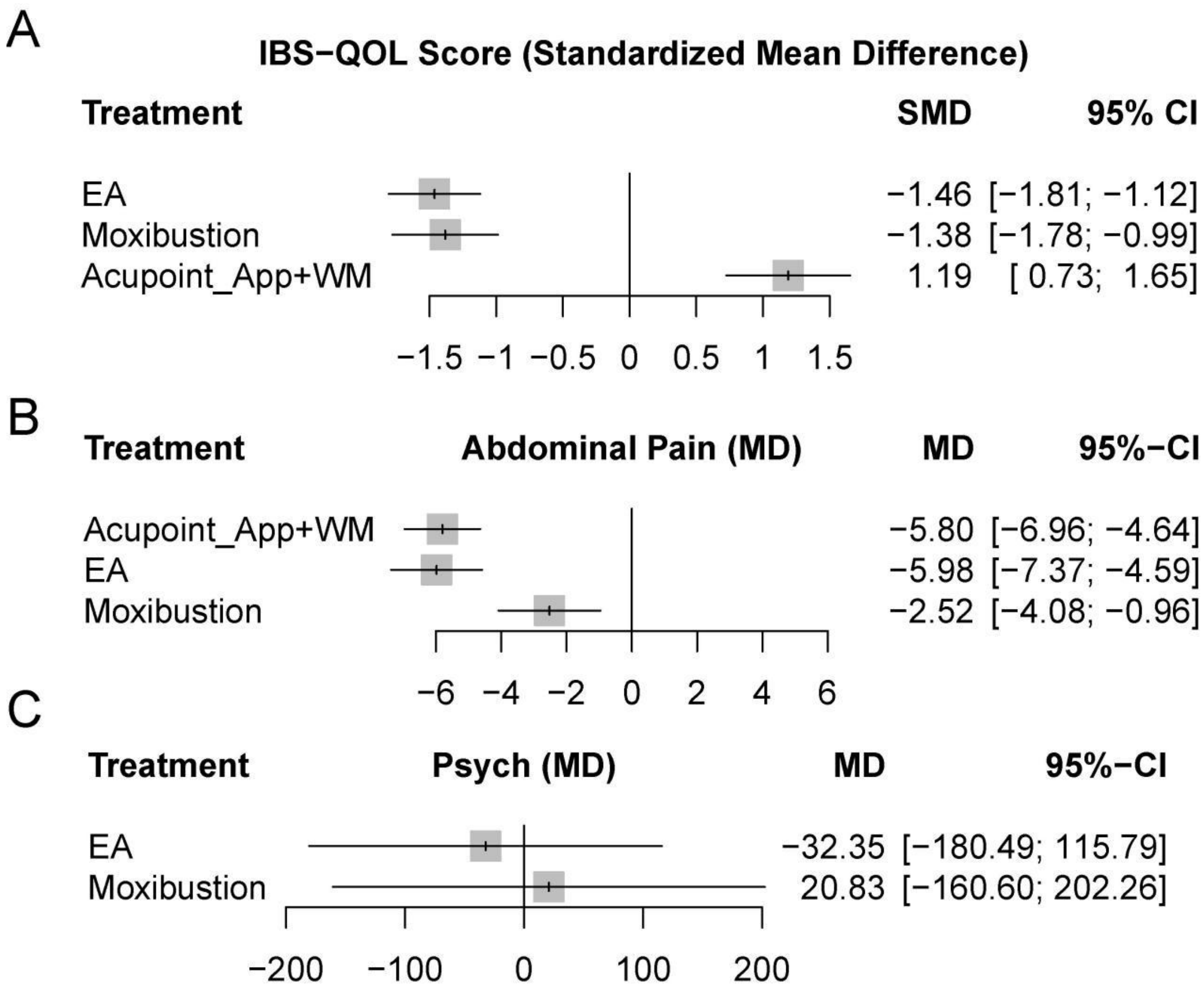

Supplementary Figure 1. Network meta-analysis results for secondary outcomes.

(A) Forest plot of interventions for IBS-QOL. Because IBS-QOL is originally a higher-is-better outcome, the direction of the scale was harmonized before synthesis; therefore, negative SMD values indicate greater improvement in quality of life.

(B) Forest plot of interventions for abdominal pain scores. Negative MD values indicate greater reduction in abdominal pain and therefore favor the intervention.

(C) Forest plot of interventions for psychological status scores, including SAS/SDS where applicable. Negative MD values indicate greater improvement in psychological symptoms and therefore favor the intervention.

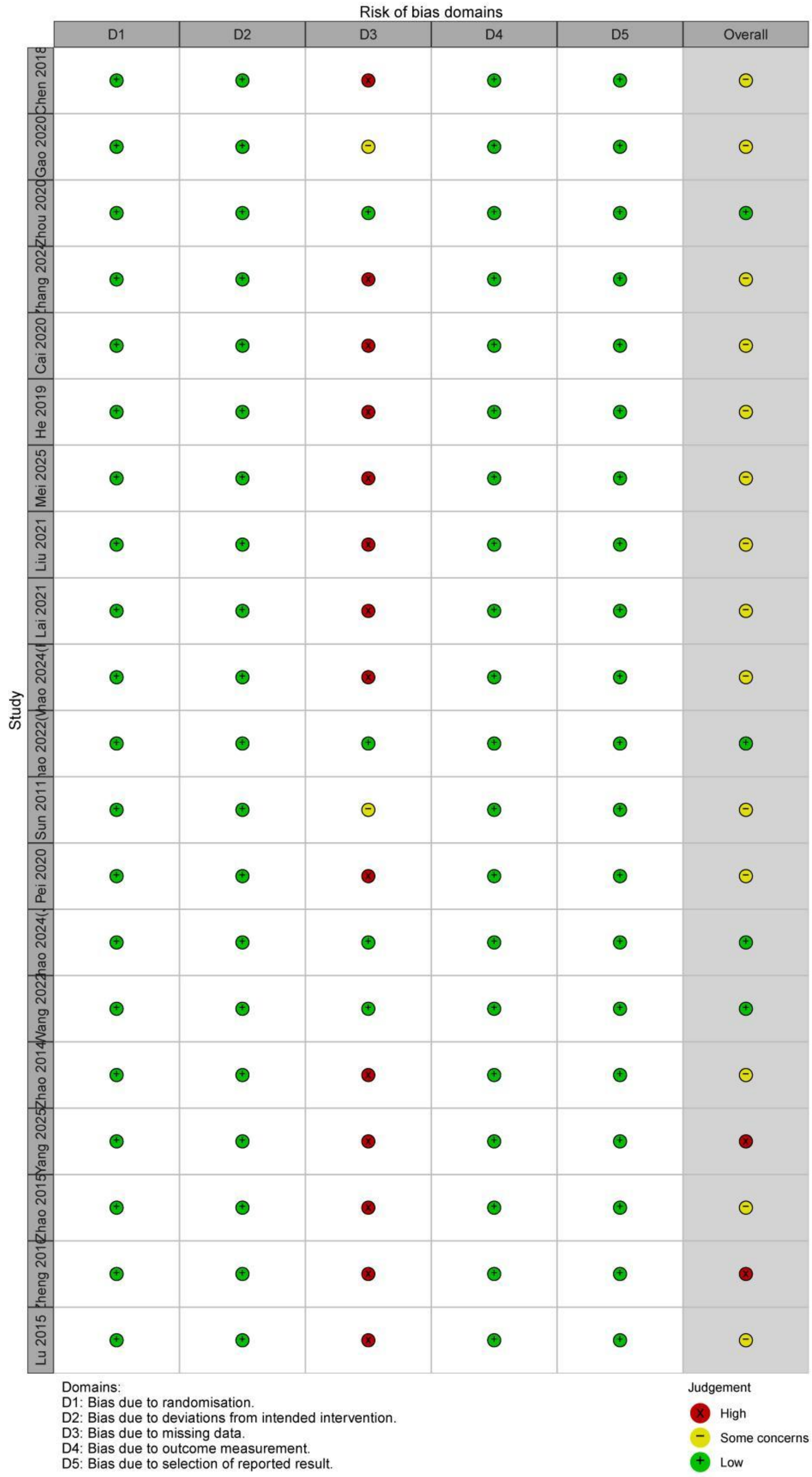

Supplementary Figure 2. Traffic light plot of risk of bias for individual studies.

Each entry represents the risk of bias assessment for each included study across five domains (D1-D5) according to the Cochrane Risk of Bias 2.0 (RoB 2.0) tool. Green, yellow, and red circles represent "Low risk," "Some concerns," and "High risk," respectively.

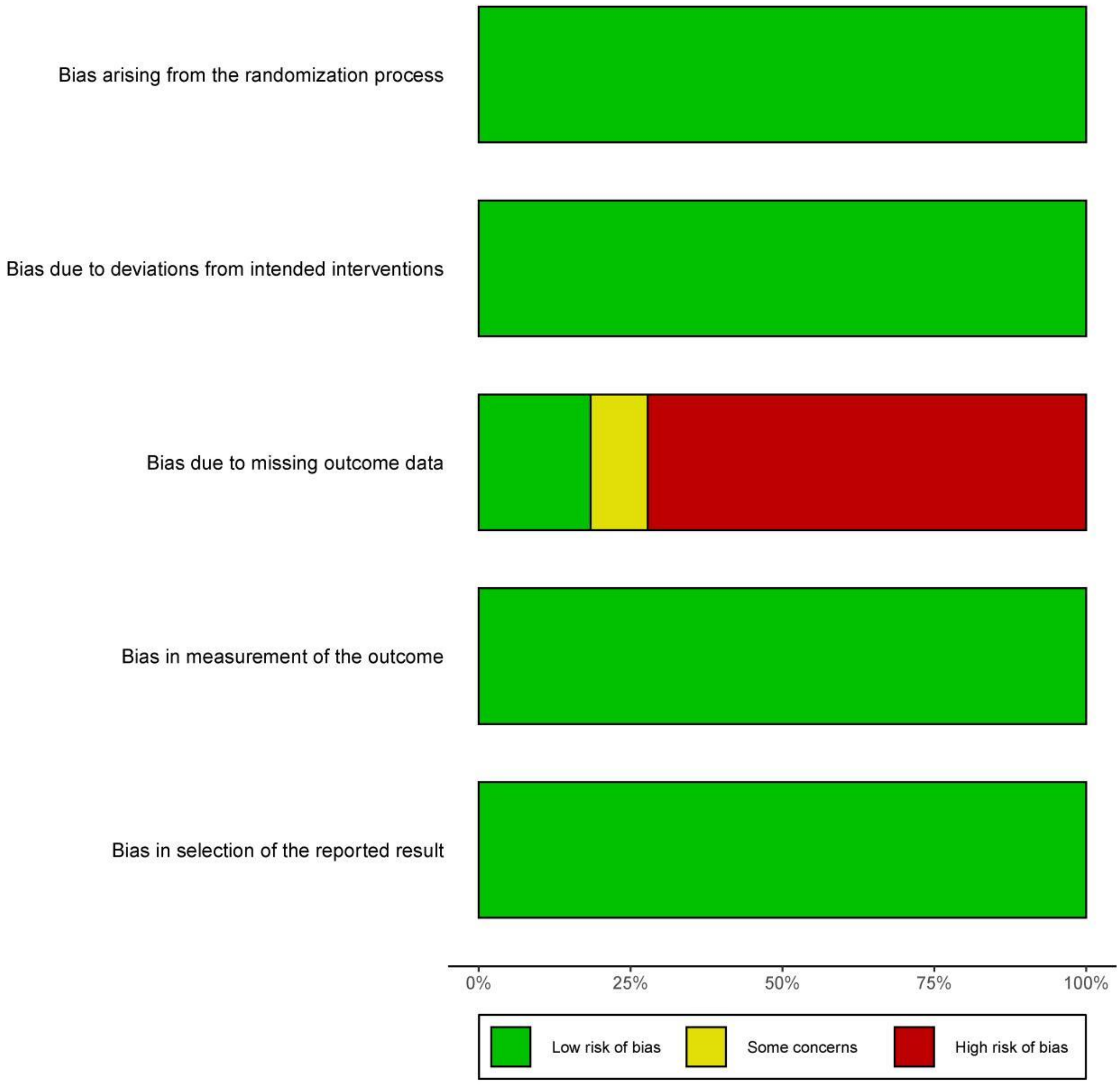

Supplementary Figure 3. Summary plot of risk of bias.

The bar chart provides an overview of the percentage of studies graded as low risk, some concerns, or high risk for each bias domain across the entire evidence base.

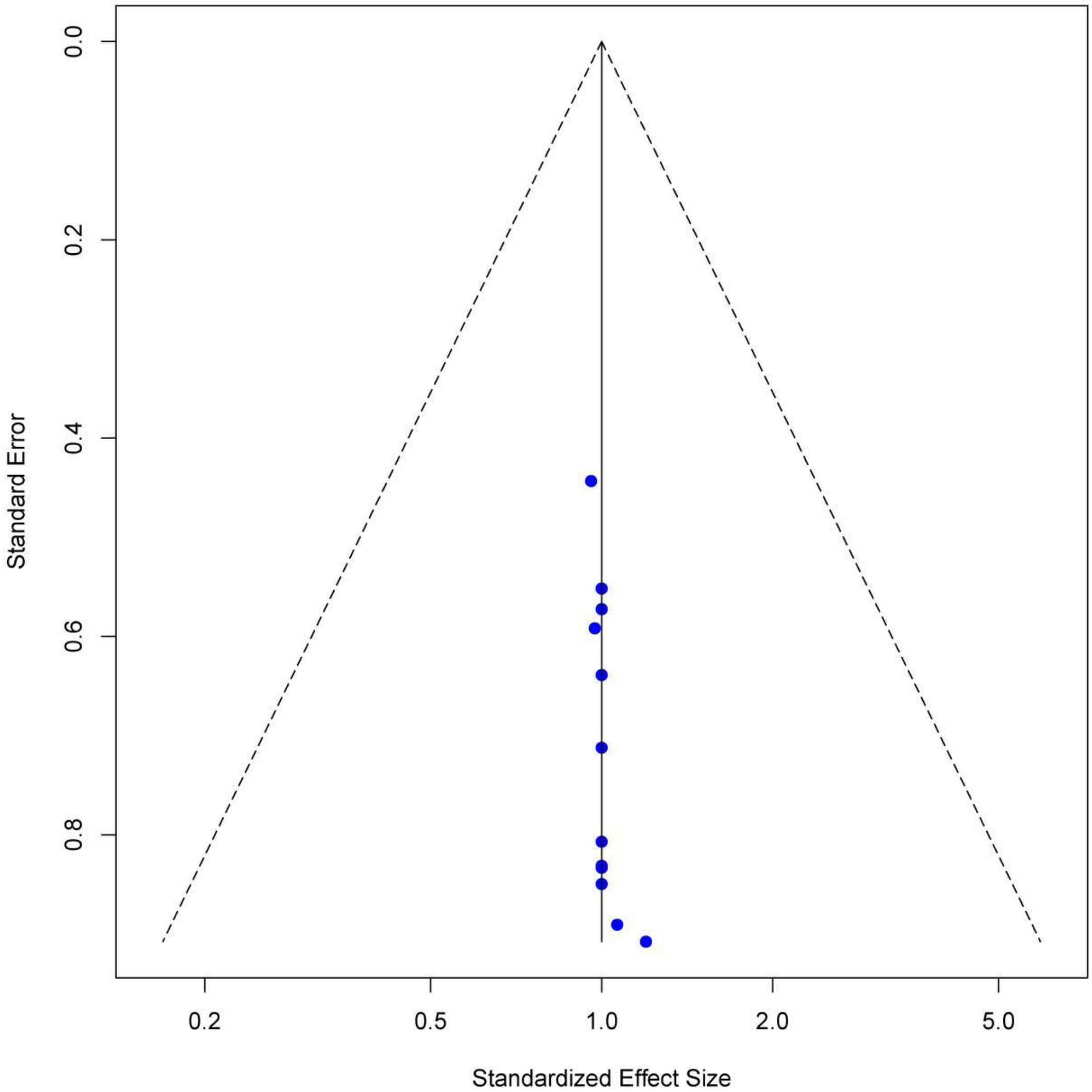

Supplementary Figure 4. Comparison-adjusted funnel plot for publication bias.

The funnel plot assesses potential publication bias for the primary outcome (Total Effective Rate). The symmetry of the dots around the vertical line suggests the absence of significant small-study effects or publication bias.
